# Fasudil, an inhibitor of Rho-associated coiled-coil kinase, improves cognitive impairments induced by smoke exposure

**DOI:** 10.18632/oncotarget.12853

**Published:** 2016-10-24

**Authors:** Deng Xueyang, Ma Zhanqiang, Ma Chunhua, Hao Kun

**Affiliations:** ^1^ Department of Pharmacology of Chinese Materia Medica, China Pharmaceutical University, Nanjing 210009, China; ^2^ Key Laboratory of Drug Metabolism and Pharmacokinetics, China Pharmaceutical University, Nanjing 210009, China; ^3^ Central Laboratory, Nanjing Municipal Hospital of T.C.M, The Third Affiliated Hospital of Nanjing University of T.C.M, Nanjing 210001, China

**Keywords:** fasudil, smoke exposure, cognitive impairment, Rho/ROCK pathway

## Abstract

The current study was designed to investigate the pathological changes in brain induced by smoke exposure, and explore whether fasudil could alleviate these impairments.

Adult C57BL/6 mice were exposed to tobacco smoking for four months, and fasudil was treated from the third months. To investigate lung injuries, the immunohistochemistry of lung tissue, immune cell infiltrations, cytokine productions in bronchoalveolar lavage (BAL) fluid, and seurm inflammatory cytokines were evaluated. To investigate cognitive impairments, Morris water maze test, hippocampal inflammatory cytokines and Rho associated signaling pathways were evaluated.

Our findings showed fasudil administration inhibited the inflitration of inflammatory cells (macrophages, neutrophils, and lymphocytes), suppressed the production of inflammatory cytokines both in the BAL fluid, serum, and hippocampus. Further, fasudil significantly improved the spatial learning and memory impairments and reduced the elevation of hippocampal inflammatory cytokines induced by tobacco smoking. Of note, expressions of RhoA, ROCK1, ROCK2, caspase-3, caspase-9, bax and the phosphorylation of NF-κBp65 were increased accompanying the smoke exposure-induced cognitive impairments, which were significantly inhibited by fasudil treatment as indicted in western blot and immunohistochemistry analysis.

Our results showed that fasudil exhibited protective effects on smoke exposure induced cognitive deficits which might involve with the regulation of Rho/ROCK/NF-κB pathways. Further studies are warranted before clinical application of fasudil.

## INTRODUCTION

Tobacco smoking is a worldwide health epidemic that is responsible for about 5 million deaths [1].Tobacco smoke exposure has been implicated in causing deleterious effects on the development of multiple organ systems [2], including the respiratory, nervous, and cardiovascular systems. Of note, detrimental effects of chronic smoke exposure on central nervous system leaded to a range of neuropsychiatric diseases such as depression, multiple sclerosis, schizophrenia, alcoholism, and cognitive impairments. Several studies have investigated the impacts of smoking on cognitive function. Researches on maternal smoking on cognitive performance in children have shown that maternal smoking is a predictor for cognitive impairments, whether measured as IQ scores or academic achievements [3].The study enrolled 30,000 subjects performed by Nichols and Chen reported that learning difficulties were associated with maternal smoking [4]. Numerous animal studies have investigated the long-term implications of cigarette smoke exposure on cognitive functions, which shown that chronic mid-gestational nicotine exposure resulted in remarkably decreased performance in adult mice in the radial-arm maze and Morris water maze tests [5], and nicotine administration resulted in an obvious effect on the mean performance of nicotine-treated rats compared to control-treated rats during the acquisition phase in the radialarm maze [6].

Increasing evidence suggests that inflammation play a crucial role in the pathobiology of smoke-exposure-induced cognitive dysfunctions. Inflammatory cytokines comprising a heterogeneous group of polypeptide mediators activate the immune systems [7].Inflammatory cytokines in the brain are toxic to neurons and may induce neuronal damages in several ways. Tumor necrosis factor alpha (TNF-α) and interleukins (ILs) produced by overactivated microglia and astrocytes cause proapoptotic and synaptotoxic effects, which are extreme detrimental to neurons [8]. Chronic cigarette smoke exposure is a serious inflammatory stimulus that induces the excess production of inflammatory cytokines, and causes deleterious effects on the nervous and respiratory systems. Therefore, inhibiting cigarette smoke-induced production of inflammatory cytokines is an attractive strategy in the development of drugs for cognitive function improvements.

Rho kinase (ROCK) is a well-known downstream effector of the small GTPases, which is closely involved in the production of inflammatory molecules [9]. Rho/ROCK signaling is believed to be implicated in the development of neuroinflammations and neuropathology of cognitive dysfunctions [10].ROCK also plays a crucial role in cell migration, reactive oxygen species (ROS) formation, and apoptosis [11, 12]. It was reported that ROCK inhibitor improved cognitive functions in rats [13], and non-steroidal anti-inflammatory drugs improved the dementia by inhibiting the activation of ROCK. Thus, ROCK inhibition is beneficial to the suppression of the development of neuroinflammation and further neuronal damages [14]. Fasudil is an inhibitor of Rho-associated coiled-coil kinase and has been demonstrated as a promising neuroprotective agents. Patients treated with fasudil within 48 h of onset of ischemic stroke improved their clinical outcomes [15].Studies have reported that fasudil protected against β-Amloid-induced neurodegeneration in animal models [16]. However, the effects of fasudil on smoke exposure-induced cognitive dysfunctions have not been reported before. Thus, in the present study, we carried out a set of *in vivo* tests to investigate the cognitive improvement effects of fasudil in a cigarette smoke exposed animal model and explored the possible mechanisms.

## RESULTS

### Fasudil improved cigarette smoke-induced learning and memory impairments

**Figure 1:**
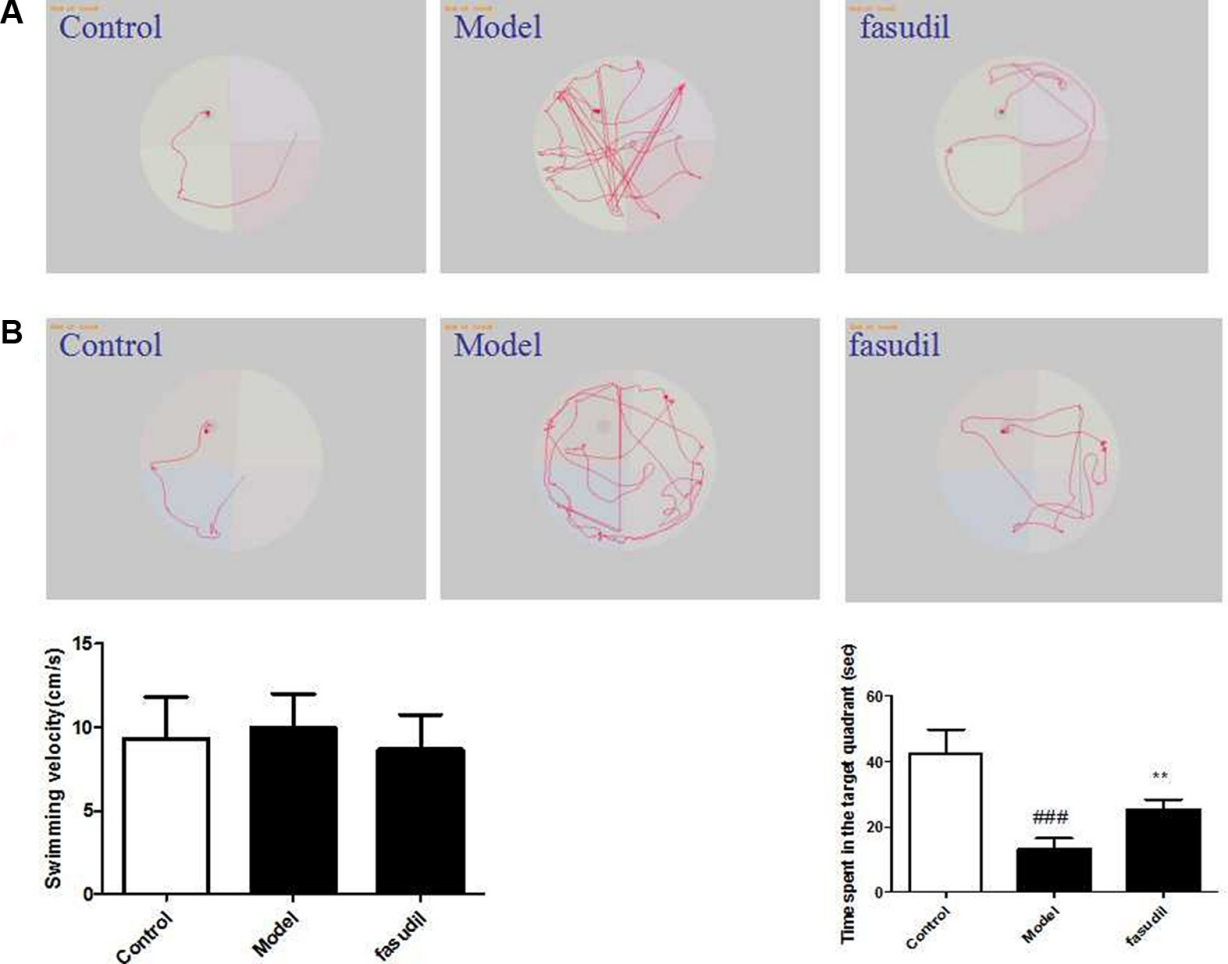
Fasudil improved cigarette smoke-induced learning and memory impairments (**A**) Representative searching strategy of rats in the third trial on the first day. (**B**) Representative searching strategy of rats in the third trial on the fifth day. Control: Normal mice; Model: the smoke exposed mice; fasudil: the smoke + fasudil (5 mg/kg) mice The data was presented as mean ± SEM, Compared with sham: ^#^*P* < 0.05, ^##^*P* < 0.01; Compared with vehicle:*P < 0.05, **P < 0.01.

As shown from Figure 1, the mean escape latency was decreased after 5 training days among all the groups. Smoke-exposed mice exhibited significantly higher escape latency than those absent from smoke stimulation, while fasudil (5 mg/kg) treatment also decreased this period. The time spent in the target quadrant increased after training which revealed the degree of memory consolidation. Mice treated with fasudil (5 mg/kg) spent more time on the target quadrant compared with the model group. Mice exposed to cigarette smoke had significant impairment in spatial learning ability as indicted from the longer escape latency in comparison with that of the control mice. While treatment with fasudil (5 mg/kg) notably shortened the escape latency compared with that in model group.

### Fasudil inhibited cigarette smoke-induced inflammation in BAL fluid

**Figure 2:**
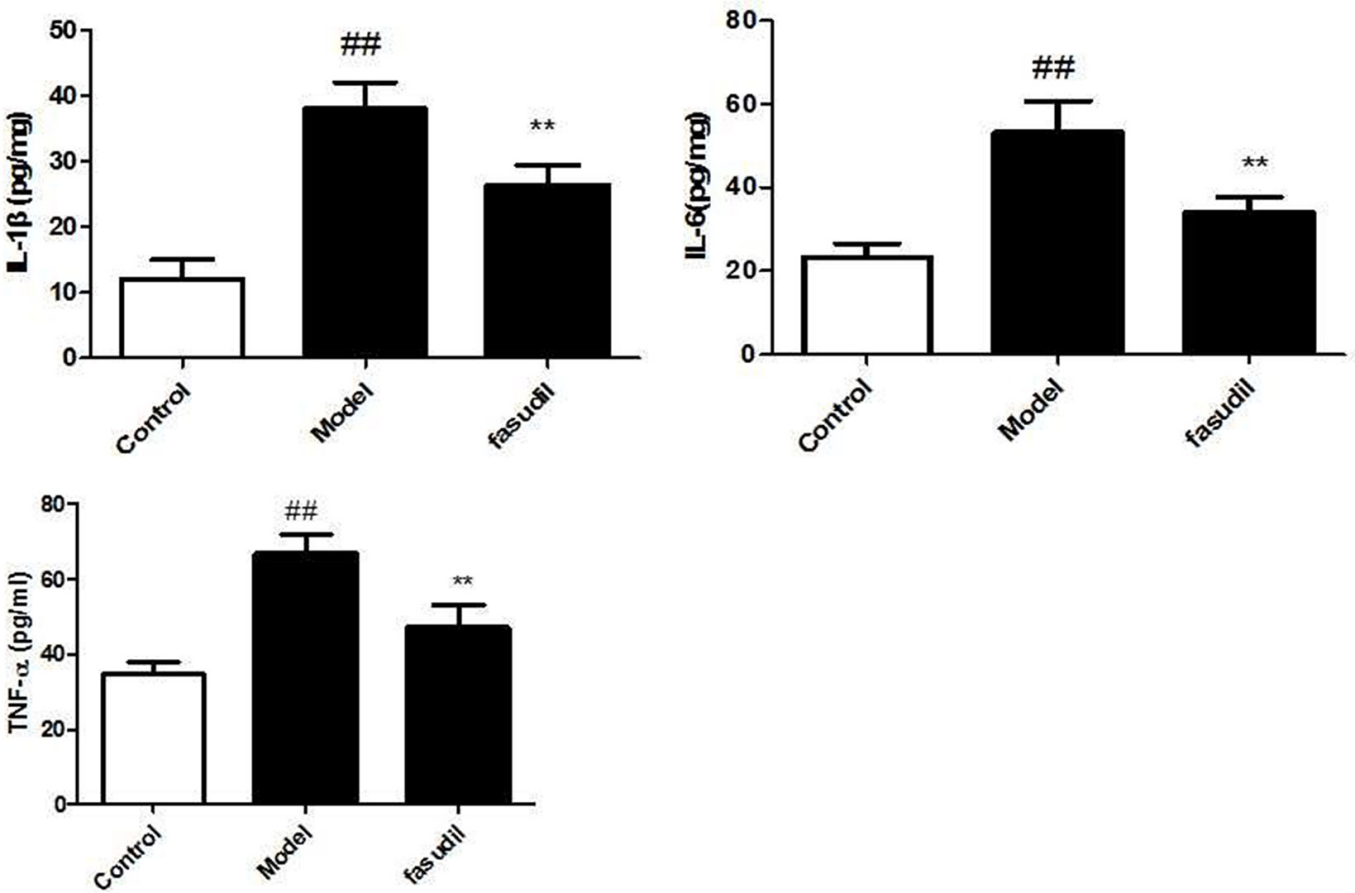
Fasudil inhibited cigarette smoke-induced inflammation in BAL fluid Control: Normal mice; Model: the smoke exposed mice; fasudil: the smoke + fasudil (5 mg/kg) mice The data was presented as mean ± SEM, Compared with sham: ^#^*P* < 0.05, ^##^*P* < 0.01; Compared with vehicle:*P < 0.05, **P < 0.01.

As shown from Figure 2, The levels of inflammatory cytokines in the BAL fluid were determined by ELISA kits. Smoke stimulation increased the levels of IL-1β, IL-6 and TNF-α in BAL fluid. Fasudil administration significantly decreased the concentrations of these inflammatory cytokines in BAL fluid compared with those in the model group.

### Fasudil inhibited cigarette smoke-induced infiltration of inflammatory cells in lung tissue

**Figure 3:**
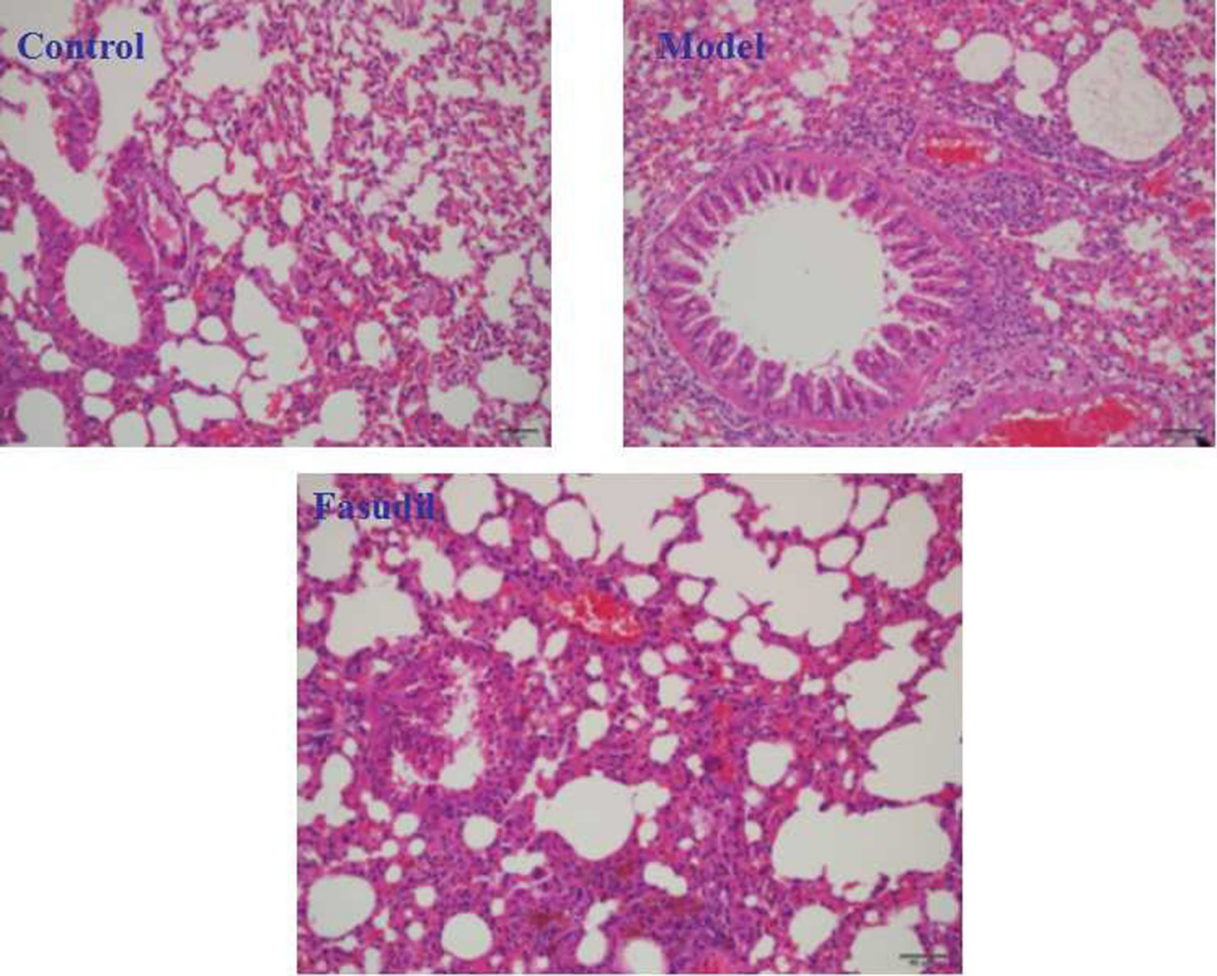
Fasudil inhibited cigarette smoke-induced infiltration of inflammatory cells in lung tissue Control: Normal mice; Model: the smoke exposed mice; fasudil: the smoke + fasudil (5 mg/kg) mice The data was presented as mean ± SEM, Compared with sham: ^#^*P* < 0.05, ^##^*P* < 0.01; Compared with vehicle:*P < 0.05, **P < 0.01.

As shown from Figure 3, the lung histology of experimental mice exposed to cigarette smoke were analyze from H&E staining. The cigarette smoke-induced model group exhibited severe infiltration of inflammatory cells both in the bronchial and peribronchial layers compared with the air group. Airspace enlargements were also observed in smoke-exposed model group. In contrast, fasudil administration showed a weakened degree of inflammatory damage and lessened airspace compared to the model group. Thus, fasudil significantly abrogated the smoke stimulation-induced lung injury.

### Fasudil inhibited the cigarette smoke-induced elevation of cytokines in serum and hippocampus

**Figure 4:**
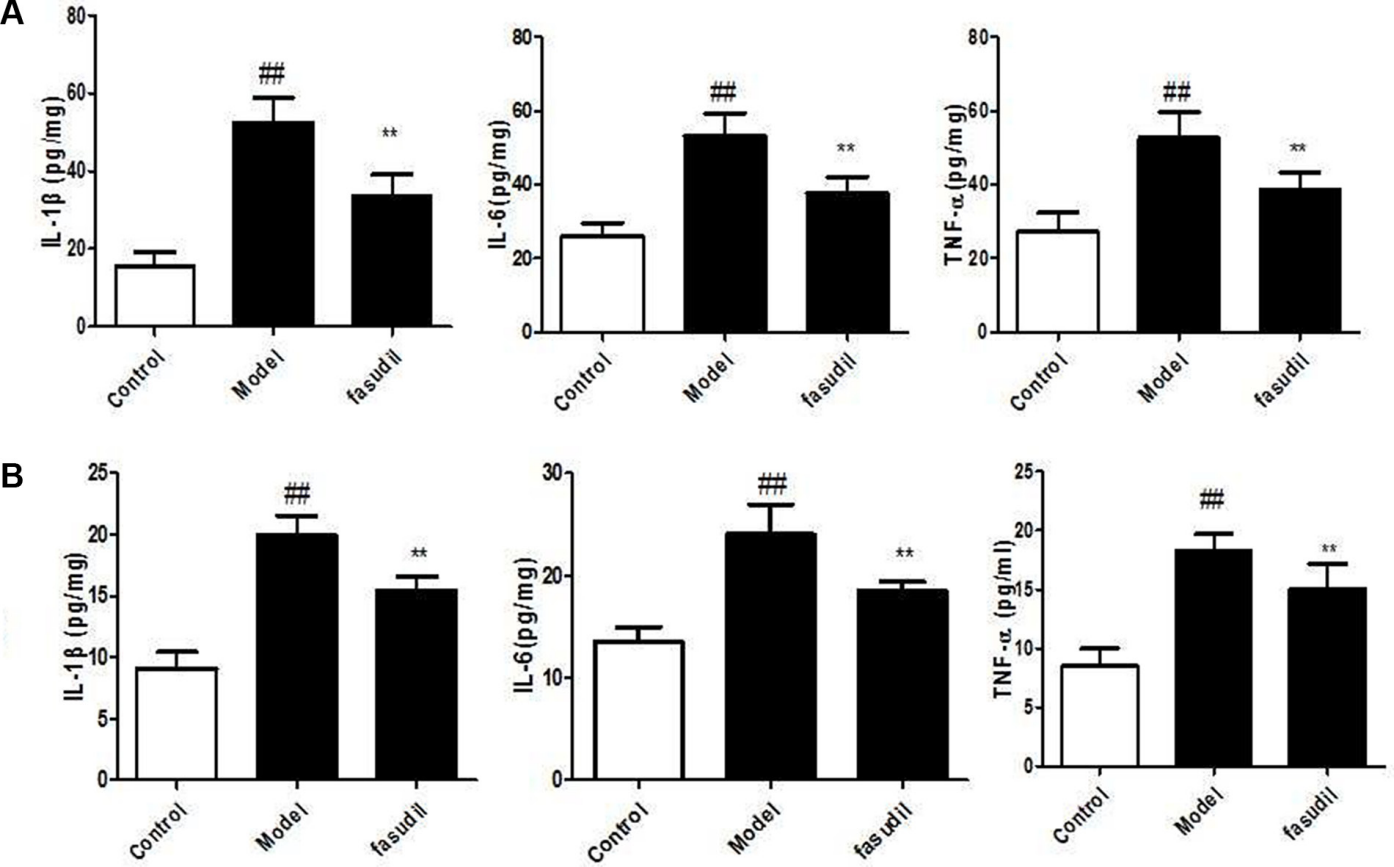
Fasudil inhibited the cigarette smoke-induced elevation of cytokines in serum (A) and hippocampus (B) Control: Normal mice; Model: the smoke exposed mice; fasudil: the smoke + fasudil (5 mg/kg) mice The data was presented as mean ± SEM, Compared with sham: ^#^*P* < 0.05, ^##^*P* < 0.01; Compared with vehicle:*P < 0.05, **P < 0.01.

To investigate whether fasudil also altered the cytokine responses in the serum and brain, the levels of IL-1β, IL-6, TNF-α in the serum and brain were determined after smoke exposure. As show in Figure 4, the levels of IL-1β, IL-6, TNF-α in serum and hippocampus were increased in mice exposed to cigarette smoke. Nevertheless, mice treated with fasudil exhibited decreased levels of inflammatory cytokines compared to the air-exposed mice. Thus, fasudil significantly decreased the secretion of IL-1β, IL-6, TNF-α in serum and hippocampus.

### Fasudil inhibited Rho-mediated inflammatory signalings in hippocampus

**Figure 5:**
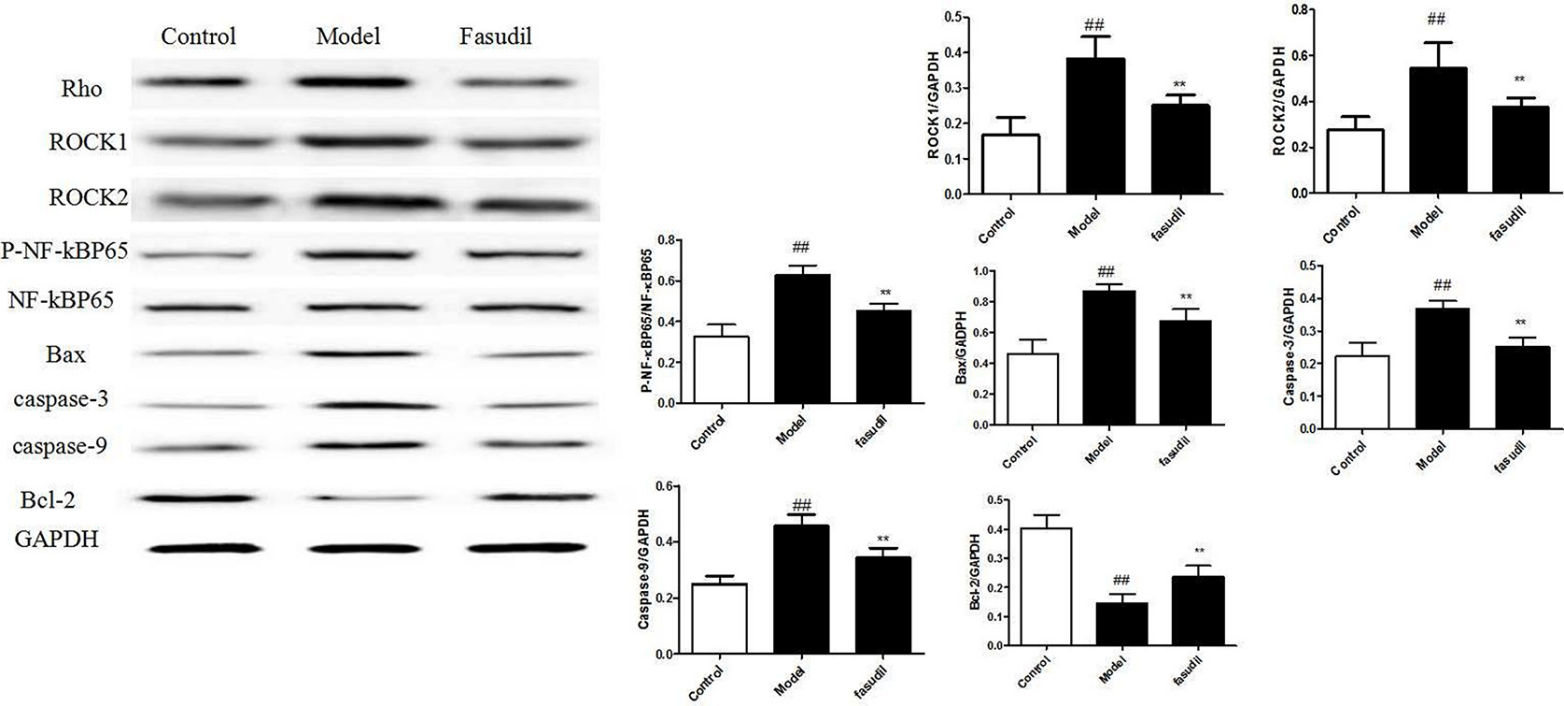
Fasudil inhibited Rho-mediated inflammatory signalings in hippocampus by Western Blot Control: Normal mice; Model: the smoke exposed mice; fasudil: the smoke + fasudil (5 mg/kg) mice The data was presented as mean ± SEM, Compared with sham: ^#^*P* < 0.05, ^##^*P* < 0.01; Compared with vehicle:*P < 0.05, **P < 0.01.

**Figure 6:**
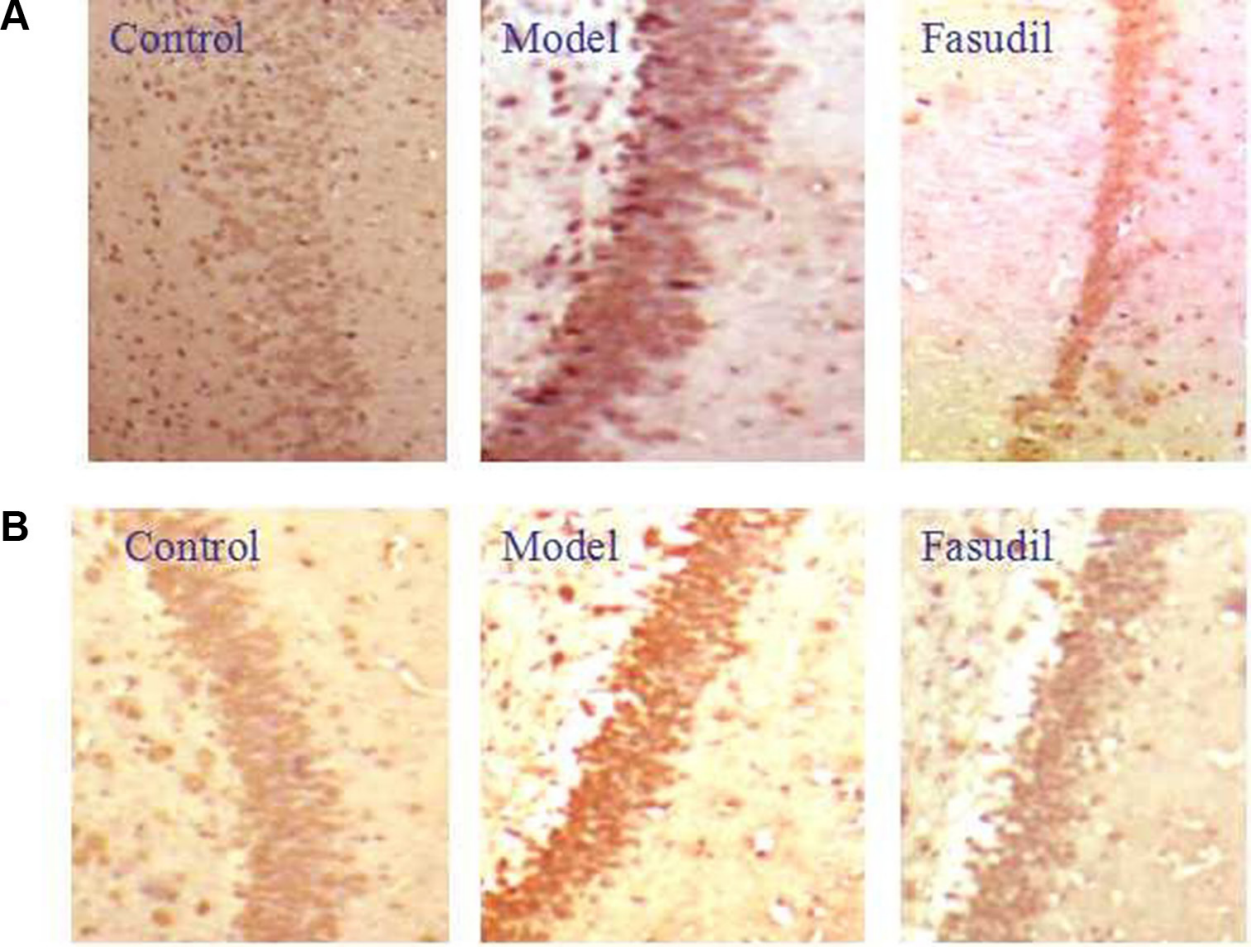
Fasudil regulated Rho-mediated inflammatory signalings in hippocampus by immunohistochemistry (**A**) immunohistochemistry of Rho; (**B**) immunohistochemistry of P-NF-κBP65 Control: Normal mice; Model: the smoke exposed mice; fasudil: the smoke + fasudil (5 mg/kg) mice

In order to characterize the intracellular signaling pathway responsible for the neuroprotective effects of fasudil in cigarette smoke-induced cognitive impairments, the Rho-mediated inflammatory signaling and NF-κB pathway were tested. The data depicted in Figure 5. demonstrated the highly expressed Rho, ROCK1, ROCK2, p-NF-κBp65, caspase-3, caspase-9 and bax in smoke-stimulated mice, while the administration of fasudil (5 mg/kg) dramatically decreased their expressions in varying degrees. The expression of Bcl-2 was suppressed after the cigarette smoke exposure, while chronic fasudil administration restored this alterations. Results obtained from immunohistochemistry (Figure 6) indicted the increased expressions of Rho, p-NF-κBp65 in smoke-exposed mice, fasudil reduced the expressions of Rho, p-NF-κBp65, which further confirmed the involvement of inflammation pathway in smoke-induced cognition deficits.

## DISCUSSION

Up to now, fasudil is the only ROCK inhibitor approved for human use, and is allowed to treat and prevent cerebral vasospasm after subarachnoid hemorrhage [17]. Meanwhile, fasudil is a clinical safe agent and no serious side effects have been found from post-marketing surveillance studies. The proposed protective mechanisms of ROCK inhibition include inflammatory responses suppression through mediating expressions of cytokines and NADPH oxidase, and activating the NF-κB pathway. Based on the overall promising investigation showing beneficial effects of fasudil in a variety of central nervous system diseases, considerable interest and attentions have been devoted to investigate neuroprotective effects of fasudil in more related diseases, including cognitive deficits. In our present study, the results showed that cigarette smoke activated inflammatory responses, and fasudil (5 mg/kg) administration inhibited the inflammatory responses both in lung and brain and improved learning and memory deficits by suppressing the Rho-mediated inflammatory and apoptosis signalings.

Cigarette smoke stimulation involved with diverse alterations in biological and physiological processes. Epidemiological data from multiple sources clearly indicated that chronic cigarette smoke exposure profoundly affected the functions of nervous and respiratory systems. Animal studies have indicated that chronic exposure to nicotine via tobacco smoke could compromise lung functions, and affected the learning and memory abilities. In our present study, cigarette smoke-exposed mice obviously exhibited the phenotype of lung inflammation, as indicted by increased numbers of inflammatory cells (macrophages, neutrophils, and lymphocytes) and inflammatory cytokines in BAL fluid, and augmented infiltration of inflammatory cells in bronchial and peribronchial layers in lung tissues. These were consistent with the clinical data, which showed the presence of significantly higher levels of IL-6 in BAL fluid and serum in smokers, compared with healthy controls [18]. As been documented, inflammatory cytokines in the brain are toxic to neurons and may induce neuronal damages in several ways [19]. Hence, the concentrations of hippocampal cytokines were measured to evaluate the severity of neuroninflammation injury of smoke-exposed mice. The acquired data suggested that cigarette smoke stimulation caused brain inflammatory responses as indicated by the elevated level of IL-1β, TNF-α and IL-6 in hippocampus of cigarette smoke-exposed mice. Chronic fasudil (5 mg/kg) administration for two months inhibited the overproduction of cytokines and lessened the inflammatory impairments to learning and memory capabilities.

To further illustrate the molecular mechanisms related to the brain recovery abilities of fasudil on mice with smoke-induced cognitive impairments, western blot and immunohistochemistry analysis were adopted to determine the protein expressions of Rho-mediated inflammatory signaling. ROCK α/ROCK2 and ROCK β/ROCK1 are two isoforms of ROCK, which have been identified as the effector of Rho and play important roles in the pathogenesis of nervous and mental diseases. Excessive ROCK activities impair neuron survival pathway, in part, by promoting pro-inflammatory pathways including enhanced expression of adhesion molecules and increased production of inflammatory cytokines [20].The acquired data suggested that smoke stimulation increased the expressions of RhoA, ROCK1 and ROCK2, while fasudil dramatically reversed these alterations. As the downstream target of ROCK, NF-κB signaling has been widely reported to be involved in inflammatory responses [21]. Our results further demonstrated that NF-κB signaling was significantly activated in smoke-exposed mice. In quiescent cells, NF-κB activities are suppressed by interacting with IκB in cytoplasm [22]. Once simulated, IKKα/β phosphorylate induces IκBα degradation through the IκB kinase complex and allows NF-κBp65 to translocate to nuclear. In the present study, we discovered that fasudil abrogated the smoke-induced phosphorylation of NF-κB p65. Thus, it was probably that fasudil improved learning and memory capabilities in mice through inhibiting the activation of NF-κBp65 which consequently decreased the gene expression of inflammatory cytokines and alleviated the severe inflammatory detriments. ROCK mediated inflammatory signaling induced caspase activation which ultimately led to cell apoptosis [23]. Our results showed that fasudil regualted apoptosis related proteins bas, Bcl-2, caspase-9 and caspase-3 and inhibited mitochondrial apoptosis pathway.

In summary, we found that cigarette smoke induced lung injury accompanied with the decreased cognitive functions, of which inflammation might be their common underling mechanisms. Fasudil exhibited protective effects on smoke exposure induced cognitive deficits which might involve the regulation of Rho/ROCK/NF-κB pathways. This may offer opportunities for fasudil in the preventive interventions of nervous and respiratory diseases. Further studies are warranted before clinical application of Fasudil.

## MATERIALS AND METHODS

### Reagents and kits

Fasudil was purchased from Tianjin Chase Sun Pharmaceutical. Co., Ltd (Tianjin, China). Enzyme-linked immunosorbent assay (ELISA) kits of IL-1β, IL-6 and TNF-α were obtained from Nanjing KeyGEN Biotech. CO., LTD. (Nanjing, China). Primary antibodies against RhoA, ROCK1, ROCK2, p-NF-κBp65, NF-κBp65, caspase-3, caspase-9, bax and Bcl-2 were produced by Cell Signaling Technology (Danvers, USA).

### Animals

C57BL/6 male mice (7 to 8 week old) were obtained from the Experimental Animal Center of China Pharmaceutical University (Nanjing, China). Mice were maintained in an animal center, with free access to food and water under standard laboratory condition. Experiments were conduced strictly in accordance with the Provision and General Recommendation of Chinese Experimental Animals Administration Legislation. All animal experiments were followed with protocols approved by Medicine Animal Care and Use Committee of China Pharmaceutical University.

### Experimental design

The C57BL/6 mice were exposed to cigarette smoke 1 hour once a day for four months using 3R4F Kentucky reference cigarettes (University of Kentucky, Lexington, KY, USA) by a standardized smoking procedure. Animals treated with fasudil began from the third month. Animals were divided into three groups: the normal group (Control); the smoke exposed group (Model); the smoke + fasudil (5 mg/kg) group (fasudil). Animals in the control group were exposed to the fresh air. The mice were treated with vehicle or fasudil (5 mg/kg) via a single i.p. injection before exposure to cigarette smoke. The Morris water maze test was performed twenty-four hours after the last smoke exposure. After that, the blood samples were collected, and the BAL fluid, lung and brain tissues were prepared for further study.

### Morris water maze test

A modified Morris water maze test was performed in C57BL/6 mice of each group. The test was performed 1 h after the last fasudil administration. In brief, it was conducted in a circular pool, with 180 cm in diameter and 60 cm in height. A submerged round platform, 8 cm in diameter, was placed in the center of target quadrant. The position of the cues was standing still throughout the test. Mice were put into the water individually to record the period that mice needed to locate and stay on the platform within 90 s and 20 s respectively. A video camera linked to the MiniSee computerized tracking system has been adopted to record the escape latency during the test. Three trial sessions were performed daily for five consecutive days during the Morris water maze test.

### Bronchoalveolar lavage (BAL) fluid

Mice were euthanized after the behavioral experiments and the lung tissue was lavaged with cold PBS for three times. The bronchoalveolar lavage (BAL) fluid was centrifuged at 1500 g for 10 min, and the supernatants were stored at −80°C till analysis of cytokines and chemokines. Total cells, lymphocytes, neutrophils and macrophages were counted using a hematology analyzer.

### Lung histology

Lungs tissue were excised from the mice and fixed with 10% buffered formalin for histopathological evaluation. Samples were fixed with 10% neutral formalin and embedded in paraffin. Tissue were sectioned into 5-μm thickness with a microtome and stained with hematoxylin and eosin (H&E) to examine cell infiltration. Lung histology was conducted by light microscopic examinations (Nikon, Tokyo, Japan).

### Analysis of inflammatory cytokines in serum, BAL fluid and hippocampus

The concentrations of IL-1β, IL-6, TNF-α in serum, BAL fluid and hippocampus were determined by an enzyme-linked immunosorbent assay (ELISA) kits, according to the manufacturer's instructions (Nanjing KeyGEN Biotech. CO., LTD., Nanjing, China),. The results of the levels of inflammatory cytokines were expressed as pictograms per milligram protein.

### Immunohistochemistry

The expressions of Rho and p-NF-κBp65 in the hippocampus were evaluated by immunohistochemistry staining. In brief, the hippocampus tissues were embedded in paraffin and sectioned. Then, the paraffin sections were deparaffinized in xylene, rehydrated by ethanol and incubated with 3% hydrogen peroxide. Hippocampal samples were blocked with 3% BSA and incubated with respective primary antibody at 4°C overnight. After incubated with secondary and three antibodies, samples were stained with DAB and observed under a microscope.

### Western blot analysis

The hippocampus tissues were homogenized in ice-cold RIPA buffer (0.1% phenylmethylsulfonyl fluoride) and centrifugated at 12000 g for 5 min. Based on the protein concentration determined by BCA kit (Beyotime Biotechnology, Nanjing, China), samples were loaded by a SDS-polyacrylamide gel electrophoresis. After blocked with 5% fat-free milk for 2 h, the PVDF membranes were incubated with respective primary antibodies, anti-RhoA, anti-ROCK1, anti-ROCK2, anti-p-NF-κBp65, anti-NF-κBp65, anti-caspase-3, anti-caspase-9, anti-bax and anti-Bcl-2 (CST, Danvers, USA) at 4°C overnight. Immunoreactive bands were visualized by a BIO-RAD ChemiDoc XRS system and densitometric analysis were determined by Image J software (National Institutes of Health, USA).

### Statistical analysis

All data were expressed as mean ± S.E.M. Multiple comparisons were performed by analysis of variance (ANOVA) with Tukey's post hoc test by SPSS 17.0 (SPSS Inc., USA). A *P* value less than 0.05 was considered statistically significant.
